# The role of IFIH1 gene rs1990760 and rs2111485 single-nucleotide polymorphisms in generalized vitiligo predisposition

**DOI:** 10.3906/sag-1808-63

**Published:** 2019-02-11

**Authors:** Duru ONAN, Ahu YORULMAZ, Fatih Süheyl EZGÜ, Kadir Mutlu HAYRAN, Seray KÜLCÜ, Refika Ferda ARTÜZ, Başak YALÇIN

**Affiliations:** 1 Department of Dermatology, Ankara Numune Training and Research Hospital, University of Health Sciences, Ankara Turkey; 2 Department of Pediatric Genetics, Faculty of Medicine, Gazi University, Ankara Turkey; 3 Department of Preventive Oncology, Cancer Institute, Hacettepe University, Ankara Turkey

**Keywords:** Vitiligo, single nucleotide polymorphism, genes

## Abstract

**Background/aim:**

Interferon-induced helicase* (IFIH1)* is a gene locus that has been recently defined as a candidate for susceptibility to generalized vitiligo (GV). The objectives of this study were to assess the association of *IFIH1* gene, rs2111485, and rs1990760 single-nucleotide polymorphisms (SNP) with susceptibility to GV and the autoimmune diseases accompanying GV.

**Materials and methods:**

We prospectively studied GV patients and frequency-matched healthy controls by age and sex. The genotypes of the participants were determined for rs1990760 and rs2111485 SNPs of *IFIH1*. Dominant, recessive, and additive models were evaluated for each SNP adjusted for age and sex.

**Results:**

The patients and their controls were observed to be in the Hardy–Weinberg equilibrium for SNP1 (2q24.2, rs1990760, *IFIH1*, T/C) and SNP2 (2q24.2, rs2111485, *IFIH1*, G/A), respectively (all P > 0.7). For SNP1, every T allel addition was significantly associated with 1.53 times protectiveness in terms of vitiligo risk (P = 0.033). As for SNP2, every G allel addition was associated with 1.42 times protectiveness, close to statistical significance (P = 0.100). **Conclusions:** We detected that for SNP1, each T allel and for SNP2, each G allel are protective in terms of vitiligo development. Hereby, we confirmed that *IFIH1* gene locus has a role in GV susceptibility.

## 1. Introduction

Vitiligo is a common acquired disorder in which depigmented skin results from destruction of melanocytes (1). Generalized vitiligo (GV), the prevalent form of the disease, results from the interaction among multiple genetic and environmental factors that ultimately cause autoimmune destruction of melanocytes in affected regions (2). Vitiligo occurrence in other members of patients’ families confirms the role of genetic factors in pathogenesis of the disease (3). 

Vitiligo pathogenesis involves innate immune responses triggered by environmental and cell-intrinsic factors that cause melanocyte stress. Damaged melanocytes release damage-associated molecular patterns (DAMPs), such as reactive oxygen species (ROS) and heat shock proteins (HSPs), that function as ligands for innate pattern recognition receptors (PRRs). Activation of PRRs by stress-induced DAMPs initiate inflammation that drives melanocyte destruction (4). 

Vitiligo often accompanies other autoimmune diseases, including type 1 diabetes mellitus (T1D), autoimmune thyroid disease, rheumatoid arthritis (RA), alopecia areata, adult-onset autoimmune diabetes mellitus, inflammatory bowel disease, pernicious anemia, autoimmune gastritis, psoriasis, systemic lupus erythematosus (SLE), discoid lupus, Guillain–Barré syndrome, myasthenia gravis, Addison’s disease, linear morphea, and Sjögren’s syndrome (2,5). This epidemiologic association can be seen also in the close relatives of GV patients, proposing that common genes lie behind predisposition to this group of diseases (6). 

*IFIH1* (interferon-induced helicase), also called *MDA-5 *(melanoma-differentiation-associated gene 5), is a locus in the innate immunity viral RNA receptor gene region on chromosome (2q24.3) (Gene ID: 64135) (7). The natural immune system senses viral infection by distinguishing a variety of viral components and thus initiates antiviral responses (8). *IFIH1 *encodes an RNA helicase that mediates the natural immune system’s antiviral interferon response through viral double-stranded (ds)RNA recognition (8,9). By functioning as a PRR, *IFIH1* also activates innate immune responses on binding DAMPs that are not associated with pathogens, hereby giving way to autoimmune triggering (10). 

*IFIH1* has been reported to be associated with several autoimmune diseases such as T1D, Graves’ disease, multiple sclerosis, psoriasis, and possibly lupus (u28ef,u28f2); it has also been recently defined as a candidate for susceptibility to GV (u28f3). No population-based, randomized controlled study has been reported since then to confirm the association between *IFIH1* and vitiligo. 

The research question of this study is whether *IFIH1* gene locus plays a role in GV susceptibility. Accordingly, we aimed to investigate the association of *IFIH1 *gene polymorphisms with susceptibility of GV and the autoimmune diseases accompanying GV.

## 2. Materials and methods

This gene analysis study was designed as a case-control study. Institutional Ethics Committee of our research hospital approved the project (757/2014) and informed consent was obtained from the subjects. We prospectively studied GV patients and frequency-matched healthy controls by age and sex in the Turkish population. The subjects were selected consecutively from the patients who were admitted to the Dermatology department of our hospital between January 2014 and August 2015. Patients who did not meet the term of nonsegmental vitiligo in the dermatological examination and controls with any autoimmune disease diagnosis and family history of vitiligo were excluded from the study. 

The patient group was questioned for skin phototype, age of vitiligo onset, disease duration and activity, treatment history, and repigmentation history (spontaneous/after treatment). Any other concomitant autoimmune disease, family history of vitiligo, or any other autoimmune disease were also recorded. 

### 2.1. The analysis of rs2111485 and rs1990760 single-nucleotide polymorphisms (SNPs) 

#### 2.1.1. DNA isolation 

Blood samples were collected from the patients and the controls into EDTA tubes in order to isolate the genomic DNA. The samples were transferred to a molecular analysis laboratory where the DNA isolation was carried out by using InvitrogenIprep® isolation system. The quality and the quantity of the isolated DNA was detected with Nanodrop® spectrophotometer. DNA samples were stored in eppendorf tubes that do not contain DNAse and RNAse until the time of analysis in a calibrated deep-freezer at −200 °C. 

#### 2.1.2. Amplification of the target regions

rs2111485 and rs1990760 SNPs were prepared and ordered from the ABI File Builder Software® system. Genotyping solution was diluted 20X with 1XTE buffer in order to form the study solution which was then stored at −200 °C. The analysis was performed with ABI Fast 7500 HT real-time PCR system by using a plate of 96 wells. For the amplification, 11.25 µL of genomic DNA was mixed with TaqMan® Universal PCR Master Mix (2X), 12.5 µL of No AmpErase UNG, and 1.25 µL of 20X workingstock of SNP GenotypingAssay per each well and was amplified in a thermal cycler. Thermal cycler conditions for amplification: enzyme activation at 95 °C for 10 min; 40 cycles of denaturation at 92 °C for 15 s; then 40 cycles of annealing and extension at 60 °C for 1 min. 

#### 2.1.3. Allelic discrimination and analysis

After the PCR amplification was completed, the genotypes of the participants in terms of rs2111485 and rs1990760 SNPs of *IFIH1* locus were determined by ABI 7500 Fast analysis system.

The results were analyzed by a clinical and biochemical geneticist. A precise result was obtained for at least one of the SNPs in 127 of the patients and 111 of the controls. The missing results were due to amplification failure in PCR, probably resulting from unmatched primers due to a different underlying SNP or low-quality DNA. With the completed number of patients, the study has 80% power to detect an odds ratio of 2.2 for SNP1 and 2.4 for SNP2 with 5% type-I error level as statistically significant.

### 2.2. Statistical analysis

IBM SPSS version 21 was used for data analysis. Descriptive statistics were presented by frequencies, percentages, and median (min, max) values. The patients and their controls were tested for the Hardy–Weinberg equilibrium for SNP1 (2q24.2, rs1990760, IFIH1, T/C) and SNP2 (2q24.2, rs2111485, *IFIH1*, G/A), respectively. Dominant (C/C, C/T-T/T; A/A, A/G-G/G), recessive (C/C-C/T, T/T; A/A-A/G, G/G), and additive models were evaluated for each SNP, with adjustment for age and sex using logistic regression. In the additive model, additional protectiveness of each T allele for SNP1 and each G allele for SNP2 were estimated. The association between patient characteristics and each SNP was investigated using Chi-square test. A P-value of less than 0.05 was considered to indicate statistical significance. 

## 3. Results 

There was no difference in terms of mean ages between the vitiligo patient group (40.6 ± 16.5) (range: 13–82) and the control group (43.0 ± 17.0) (range: 10–84) (P = 0.262). Similarly, no difference was detected in terms of sex between the vitiligo patient group [71 females (55.9%); 56 males (44.1%)] and the control group [64 females (57.7%); 47 males (42.3%)] (P = 0.791). The mean age of vitiligo onset was 33.5 ± 17.2 years and the mean of disease duration was 7.1 ± 9.8 years in the patient group (Table 1).

**Table 1:** Clinical characteristics of the study subjects.

Clinical characteristics	Vitiligo patients		Controls	
	n = 127	%	n = 111	%
Age (years) (mean ± SD)	40.6 ± 16.5		43.0 ± 17.0	
Sex (female/male)	71/56	55.9/44.1	64/47	57.7/43.3
Skin phototype II III IV	23 46 58	18.1 36.2 45.7		
Disease activity stable progressive regressive	35 88 4	27.6 69.3 3.1		
Treatment history (+/-)	66/61	52.0/48.0		
Family history of autoimmune disease (+/-)	38/89	29.9/70.1		
Family history of vitiligo (+/-)	43/84	33.9/66.1		

When we evaluated the skin phototype of the vitiligo patients, we found that the skin phototype IV was the most common type (45.7%; n = 58). The disease activity was stable in 27.6%, progressive in 69.3%, and regressive in 3.1% of the vitiligo patients. Only 52% (n = 66) of them had received a treatment for vitiligo before; this was in the form of topical treatment in 84.8% (n = 56) and phototherapy in 15.2% (n= 10) of the patients. Repigmentation was detected after treatment in 31.3% (n = 40) of the vitiligo patients, and in 15.6% (n= 20) of the vitiligo patients it was spontaneous. Family history of vitiligo was reported by 33.9% (n = 43) and family history of any other autoimmune disease was reported by 29.9% (n = 38) of the vitiligo patients. (Table 1)

There was at least one concomitant autoimmune disease in 28.3% (n = 36) of the vitiligo patients. In 8.6% (n = 11) of the vitiligo patients, there were two additional autoimmune diseases other than vitiligo. Autoimmune thyroid disease was the most common autoimmune disease accompanying vitiligo (18.1%; n = 23) (Table 2).

**Table 2:** Distribution of autoimmune disease types accompanying vitiligo.

Autoimmune diseases	n	%
Total	36/127	28.3
Thyroid disease	23	18.1
Pernicious anemia	9	7.0
Alopecia areata	4	3.2
Type 1 diabetes mellitus	3	2.4
Lupus	2	1.6
Bullous pemphigoid	1	0.8
Scleroderma	1	0.8
Lichen planus	1	0.8
Celiac disease	1	0.8
Idiopathic thrombocytopenic purpura	1	0.8
Rheumatoid arthritis	1	0.8
Two different autoimmune diseases	11	8.6

The study groups were observed to be in the Hardy–Weinberg equilibrium for SNP1 (P = 0.99, P = 0.65) and SNP2 (P = 0.78, P = 0.78) for the patients and the controls, respectively. 

We obtained a result for SNP1 in 114 of the patients and 108 of the controls; and for SNP2 in 98 of the patients and 85 of the controls. It was shown that, for SNP1, every T allele addition was significantly associated with 1.53 times (%95 CI:1.03-2.26) protectiveness in terms of vitiligo risk (P = 0.033). As for SNP2, every G allele addition was associated with 1.42 times (%95 CI:0.94-2.16) protectiveness, which was close to the level of statistical significance (P = 0.100) (Table 3).

**Table 3:** Estimated risks of vitiligo with the presence of polymorphisms for the two SNPs.

rs1990760	Genotype	Casen=114 (%)	Controln=108 (%)	OR	95% (CI) lower	95% (CI) upper	P
Additive	CC (ref.) CT TT	40 (35.1) 57 (50.0) 17 (14.9)	28 (25.9) 54 (50.0) 26 (24.1)	1.53	1.03	2.26	0.033
Dominant	CC (ref.) CT/TT	40 (35.1) 74 (64.9)	28 (25.9) 80 (74.1)	1.65	0.91	2.96	0.097
Recessive	CC/CT (ref.) TT	97 (85.1) 17 (14.9)	82 (75.9) 26 (24.1)	1.89	0.95	3.74	0.070
rs2111485	Genotype	Case n = 98 (%)	Control n = 85 (%)	OR	95% (CI) lower	95% (CI) upper	P
Additive	AA (ref.) AG GG	38 (38.8) 45 (45.9) 15 (15.3)	25 (29.4) 41 (48.2) 19 (22.4)	1.42	0.94	2.16	0.100
Dominant	AA (ref.) GG/AG	38 (38.8) 60 (61.2)	25 (29.4) 60 (70.6)	1.55	0.83	2.89	0.169
Recessive	AA/AG (ref.) GG	83 (84.7) 15 (15.3)	66 (77.6) 19 (22.4)	1.67	0.78	3.57	0.183

CI: confidence interval, OR: odds ratios adjusted for age and sex, SNP: single-nucleotide polymorphism.

No significant associations were observed between patient characteristics (disease activity, repigmentation, stress history at the onset of the disease or before the exacerbations, concomitant autoimmune disease, family history of autoimmune disease and vitiligo) and SNPs (all P-values >0.15) 

## 4. Discussion 

In this gene analysis study, our primary aim was to assess the role of IFIH1 gene locus in GV susceptibility. We detected an association between IFIH1 gene polymorphisms and GV susceptibility. We showed that, in terms of vitiligo development, each T allele is protective for SNP1 and each G allele is protective for SNP2. These findings reveal that IFIH1 gene polymorphisms possibly play a role in the etiopathogenesis of vitiligo. 

Although no relationship was found between family history and gene polymorphism in our study, since the reliability of the family history given by the vitiligo patients was not certain and some of the positive family history was implying distant relatives, this result might not be reflecting the actual relationship.

To identify genes involved in susceptibility to GV; candidate gene association studies, genome-wide linkage studies and recently genome-wide association studies (GWASs) have been applied in sequence according to time. So far, GWASs have discovered approximately 50 different genetic loci that contribute to vitiligo risk, some of which are shared with other autoimmune disorders that have epidemiological association with vitiligo (18). One of these gene loci *IFIH1*, previously proven to be related with several autoimmune diseases, was recently defined as a candidate for susceptibility to GV (1,10,17). 

*IFIH1* is one of the most significantly associated loci with vitiligo (1). Several common variants in *IFIH1*, the most associated variant being the rs2111485, showed highly significant association with vitiligo. A missense variant of *IFIH1*, rs35667974 (l923V), was reported to show significant conditional association independent of rs2111485. All missense variants of *IFIH1*, i.e. rs1990760 (A946T), rs3747517 (H843R), and rs35667974 (l923V), showed association with vitiligo in the independent replication study and the former two showed associations in all three GWASs (10). 

Many vitiligo susceptibility genes that control immunoregulatory and apoptotic functions are in common with those that control other autoimmune disorders. These shared genetic associations are consistent with clinical epidemiological relations of these disorders (17,18). *IFIH1*, together with nsSNP rs1990760, was defined as the strongest candidate for T1D susceptibility (7). Nejentsev et al. confirmed the association of the two common missense polymorphisms, rs1990760 (T946A) and rs3747517 (R843H), with T1D (19). Sutherland et al. confirmed *IFIH1* as a new autoimmunity locus, extending its role in susceptibility of T1D to Graves’ disease and proposed that Ala946Thr *IFIH1 *polymorphism of SNP rs1990760 is significantly associated with organ-specific autoimmune diseases, including Graves’ disease (u28f76). Gateva et al. confirmed the association of the same missense allele of *IFIH1 *(rs1990760) with SLE (14). Psoriasis was also reported to be associated with rare missense variants in the* IFIH1* gene locus (12). 

Vitiligo-associated *IFIH1* functional variants, missense SNPs rs1990760 (A946T) and rs3747517 (R843H), were reported to be protective against T1D in European individuals (7,9,19). It was proposed that rare alleles of all associated *IFIH1 *polymorphisms protect from T1D, whereas common *IFIH1 *alleles create predisposition to the disease (19). Missense rs1990760 (A946T) was proposed to be protective against SLE (20), rheumatoid arthritis (21), multiple sclerosis (10), and Graves’ disease (16) in the European and other populations. It was shown that autoimmunity-protective* IFIH1* missense variants decrease function of *IFIH1*, thus reducing the ability to bind DAMPs involved in autoimmune triggering with eventual decreased stimulation of innate immune responses (9,10,12,22). These missense variants might be downregulating the gene response by changing the gene expression.

Genome-wide association studies of vitiligo detected that the association of *IFIH1 *functional variants (missense SNPs rs2111485, rs1990760, rs3747517, rs35667974) was protective (1,10,17). Similar to the results of these GWAS studies which were performed in the European population by Jin et al. (1,10,17), we detected a protective association of the two functional variants of IFIH1 (rs1990760 and rs2111485) for vitiligo in the Turkish population. In conclusion, although the mechanisms by which *IFIH1 *polymorphisms contribute to vitiligo pathogenesis remain to be explored, it is clear that the *IFIH1* gene locus has a role in GV susceptibility. 

## Acknowledgments

This work was supported in part by the grant of “Scientific Researches Supporting Fund” of Ankara Numune Training and Research Hospital.
